# Statistical Methods for the Analysis of Food Composition Databases: A Review

**DOI:** 10.3390/nu14112193

**Published:** 2022-05-25

**Authors:** Yusentha Balakrishna, Samuel Manda, Henry Mwambi, Averalda van Graan

**Affiliations:** 1 Biostatistics Research Unit, South African Medical Research Council, Durban 4001, South Africa; 2 School of Mathematics, Statistics and Computer Science, University of KwaZulu-Natal, Pietermaritzburg 3201, South Africa; samuel.manda@up.ac.za (S.M.); mwambih@ukzn.ac.za (H.M.); 3 Department of Statistics, University of Pretoria, Pretoria 0028, South Africa; 4 Biostatistics Research Unit, SAFOODS Division, South African Medical Research Council, Cape Town 8001, South Africa; averalda.vangraan2@mrc.ac.za; 5 Division of Human Nutrition, Department of Global Health, Stellenbosch University, Cape Town 8001, South Africa

**Keywords:** food composition database, nutrient database, review, statistical methods, clustering, dimension reduction, regression

## Abstract

Evidence-based knowledge of the relationship between foods and nutrients is needed to inform dietary-based guidelines and policy. Proper and tailored statistical methods to analyse food composition databases (FCDBs) could assist in this regard. This review aims to collate the existing literature that used any statistical method to analyse FCDBs, to identify key trends and research gaps. The search strategy yielded 4238 references from electronic databases of which 24 fulfilled our inclusion criteria. Information on the objectives, statistical methods, and results was extracted. Statistical methods were mostly applied to group similar food items (37.5%). Other aims and objectives included determining associations between the nutrient content and known food characteristics (25.0%), determining nutrient co-occurrence (20.8%), evaluating nutrient changes over time (16.7%), and addressing the accuracy and completeness of databases (16.7%). Standard statistical tests (33.3%) were the most utilised followed by clustering (29.1%), other methods (16.7%), regression methods (12.5%), and dimension reduction techniques (8.3%). Nutrient data has unique characteristics such as correlated components, natural groupings, and a compositional nature. Statistical methods used for analysis need to account for this data structure. Our summary of the literature provides a reference for researchers looking to expand into this area.

## 1. Introduction

Nutrition relates food to an organism’s need for growth, metabolism, and repair. To determine optimum nutrition, knowledge of food components is required. Thus, food composition databases (FCDBs) are pivotal in any quantitative nutrition study. A FCDB or nutrient database is a compilation of the chemical composition of food and beverage items, obtained from chemical analyses, estimations from published literature, or unpublished laboratory reports [1].

One of the major applications of FCDBs is providing data for the estimation of nutrient intakes [2]. Nutritional epidemiology has been highlighted during the last few decades and has become an area of public health importance. Due to the growing demand for food consumption data, much research has been done regarding optimal and sound statistical methods for the assessment of dietary patterns within a population [3,4,5]. There also exists literature [6] and studies that describe the application of statistical methods to food composition data (FCD) itself, but most have conducted chemical analyses to obtain the nutrient data before applying statistical methods [6,7,8]. Other studies that have used data from FCDBs have combined nutritional data with that of dietary reference intakes [9], restaurant menus [10], or household income and food price data [11]. However, in the absence of chemical analyses and secondary data, the opportunities that exist for the statistical analysis of FCD alone need to be described. This makes it an area suitable for a literature review since it is best suited for identifying key characteristics of a topic and knowledge gaps [12]. This paper aims to determine what statistical methods have been directly applied to food composition databases and datasets. Similar to other reviews in nutrition that have looked at the use of machine learning in precision nutrition [13] and the use of artificial intelligence in nutrients science research [14], this review provides a summary of the literature and is a reference for researchers looking to expand into the statistical analysis of FCDBs.

## 2. Materials and Methods

### 2.1. Search Methods

The search was conducted on 8 March 2022. We searched PubMed, Web of Science, and Scopus for studies published for all years in the database. Databases were searched using the search terms ‘food composition database’ OR ‘food composition table’ OR ‘nutrient database’ OR ‘nutrient table’, present in either the article title, abstract, or keywords. Reference lists of the included articles were also scanned.

### 2.2. Study Selection

We imported all references identified through the searches into EndNote (EndNote, Clarivate Analytics, Pennsylvania, United States of America. Available at https://endnote.com, accessed on 6 August 2018) and screened the titles and abstracts of the studies. A study was included if:

- The title, abstract, or methods of the article described an application of a statistical method (mathematical formulas, models, and/or techniques).
- The data used was from a food composition database/dataset.

Studies were excluded if:

- The statistical analysis required the extensive use of secondary data such as dietary guidelines, recommended dietary intakes, cost data, data from supermarkets and restaurants, and consumption data.
- Chemical analyses, as part of the study, were conducted to obtain the nutrient values.
- An English translation of the article was unavailable.
- The article was not a primary study (i.e., reviews, commentaries, etc.).

If the titles and abstracts were unclear concerning our inclusion and exclusion criteria, the methodology section of the full text was accessed.

### 2.3. Data Extraction

Relevant data such as the year of publication, country of the data used in the analysis, primary and secondary objectives, statistical methods used for the analysis, and results of the study were extracted from the included studies.

## 3. Results

**Figure 1:**
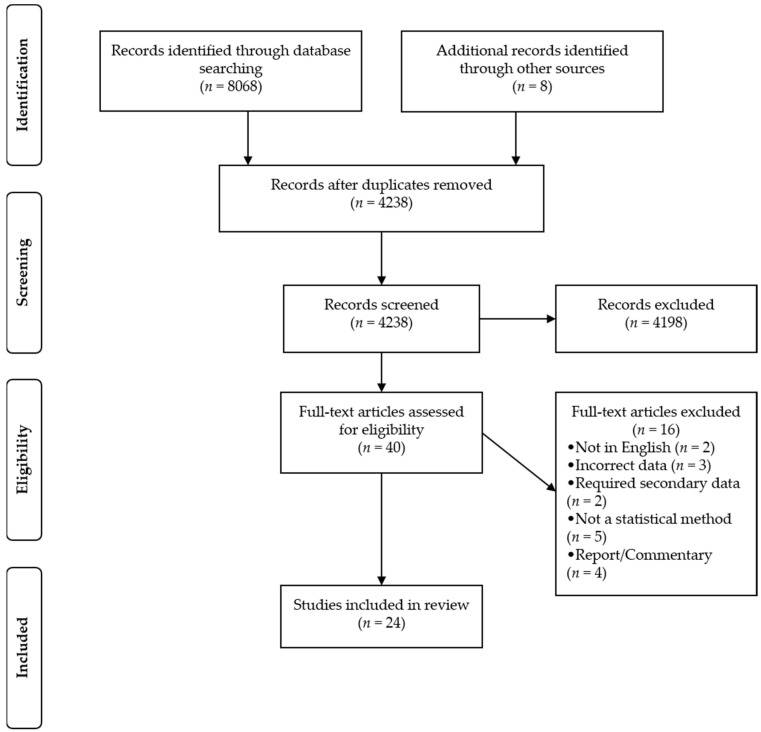
Flowchart of reviewed studies.

The search strategy yielded a total of 8076 references, distributed as follows: PubMed (*n* = 1726), Web of Science (*n* = 3190), Scopus (*n* = 3152), and other sources (*n* = 8). After the removal of duplicates, there were 4238 references, of which 24 fulfilled our inclusion criteria (Figure 1).

A description of the included studies is presented in Table 1.

The included literature spanned from 1985 to 2021, with 29.1% (*n* = 7) of the articles being published in the last 3 years. The characteristics of the included studies are shown in Table 2. The statistical methods used could be categorised into five groups: standard statistical methods, regression methods, clustering, dimension reduction techniques, and other methods. The most common statistical method used was standard statistical methods followed by clustering techniques. The objectives of the analysis could also be categorized into five themes. The studies mainly aimed to identify compositionally similar food items followed by determining associations between nutrient content and known food characteristics. We describe the application and findings for each category of statistical methods in the following sections.

### 3.1. Standard Statistical Methods

These involved the use of descriptive statistics such as frequencies and proportions, means and medians, and confidence intervals to summarize the data. Moreover, standard inferential statistics, including chi-square, Student’s *t*-test, Wilcoxon signed-rank, and Kruskal–Wallis tests, were used in an effort to generalize the findings. Eight papers utilised simple methods in their analysis of food composition databases.

Due to the attention on dietary chloride at the time, Yarbrough Al-Bander et al. [38] studied the distribution of chloride occurring naturally in uncooked food items. The results indicated high variability in the chloride and sodium content among the 216 foods analysed and a strong correlation (correlation = 0.84) between the two components. The mean difference between the chloride and sodium content (calculated as chloride content minus sodium content and measured in millimoles to allow comparison) was not statistically different from zero, indicating a high degree of coupling. These results support the need to analyse nutrients both individually and in combination with other nutrients.

Seeing the need for dieticians to be able to quickly identify foods that were either low, medium, or high in nutrient content, Khan [22] attempted to partition food items in this way. Various thresholds using measures of central tendency were tested and the results were compared. The thresholds were considered unsuitable if a ranking category contained zero food items for any nutrient, if more foods were contained in the ‘high’ category as opposed to the ‘medium’ category, and if a category contained either an extremely high count or extremely low count. Of the 20 criteria tested, 2 criteria were considered to be suitable. The criteria suggested using less than 0.5 (or 0.75) × mean, 0.5 (or 0.75) × mean to 2.5 (or 2.75) × mean, and more than 2.5 (or 2.75) × mean as cut-offs for low, medium, and high ranks, respectively. Another approach suggested that for each nutrient, the top 5%, middle 47.5%, and last 47.5% of food items be considered as the thresholds for determining high, medium, and low rankings. However, more advanced methods such as clustering algorithms may be better suited to objectively rank food items by nutrient content. By applying clustering algorithms to individual nutrients as done by Nikitina et al. [28], clusters containing foods with a low nutrient content can be separated from clusters containing a moderate nutrient content and clusters containing a high nutrient content. Thus, a natural data-driven ranking of low, medium, and high nutrient content can be found.

Three studies [19,26,35] investigated changes in the food composition of fruits and vegetables between different versions of FCDBs. All three used geometric means of each of the nutrients and compared them using the Student’s *t*-test; however, only Davis et al. [19] adjusted for moisture content. While the studies found significant changes in some nutrient components, they note that the results do not account for confounding arising from changes in chemical analyses and sampling methods and the use of mixed sources of composition data. Analysing different versions of FCDBs may be more useful in investigating the impact on consumption studies [39] rather than historical changes [40].

Regular audits to identify unlikely nutrient values are necessary to maintain a reliable FCDB. Errors could result from coding mistakes, out of range values, or laboratory analysis mistakes. Automating the auditing process will save time and resources by informing the compiler whether selective revision or further laboratory analysis is needed. Chu et al. [18] calculated and ranked coefficients of variation (CV) within each food subgroup and within each nutrient to detect outliers that may be potential errors. A rank of ‘1’ was assigned to the largest CV, ‘2’ to the second largest CV, and so on. The top two ranked CVs in each subgroup and nutrient were flagged as ‘hits’ (unlikely nutrient values) and products of the subgroup ranks and nutrient ranks that were less than or equal to 20 (since larger CVs for either subgroups or nutrients will have smaller products). The proportion of hits that were regarded as true errors by a panel of experts ranged from 1.4% to 37.6% for the various food groups. The likelihood of error detection increased 38-fold compared to manual detection and this low-cost process could help improve the accuracy of FCDBs.

Pennington and Fisher [30] determined the means and standard deviations of 24 food components in 10 previously determined subgroups (found in Pennington and Fisher [29]). The subgroups were constructed such that the nutritional composition and classification characteristics (part of a plant, colour, botanical family, etc.) for each food item were similar within each group. The subgroups exhibited unique concentrations (tested using Kruskal–Wallis ANOVA with pairwise multiple comparison procedures) of the food components and could assist the design of food frequency questionnaires (FFQs), the evaluation of dietary intake data, and the dietary guidelines provided to patients.

Nguyen et al. [27] used the Friedman and post-hoc Wilcoxon signed-rank test to determine if ‘healthier’ versions of common foods contained more sugar. By comparing fat-free, low-fat, and regular versions of the same food, it was found that the amount of sugar was higher for fat-free and low-fat versions as compared to the regular versions, despite having a lower caloric content.

### 3.2. Regression Methods

Regression methods are a powerful tool in statistics that are widely used to predict the values of dependent variables using information concerning independent variables. Three papers [21,25,37] reported applying regression methods to the FCDB. Two papers [25,37] related concepts from traditional Chinese medicine to food composition. All foods in traditional Chinese medicine are categorised into the four natures: cold, cool, warm, and hot. The purpose of these papers was to examine the association between the nutrient content of these foods and their cold-hot nature category. Liu et al. [25] utilised multiple logistic regression and found fat, carbohydrates, and selenium to be significantly associated with ‘hot’ foods while copper and iron were significantly associated with ‘cold’ foods. Xie et al. [37] additionally considered that multiple food components together may influence the cold-hot characteristics of the food. The authors fitted a multivariate ordinal logistic regression model to predict the probability of a food item being hot-, cold-, or plain-natured. Six components (folate, B6, calcium, vitamin A, and caffeine) were included as predictors in the final model. The developed model can be used to objectively classify foods as per traditional Chinese medicine. The study was limited by the lack of data for five food components, resulting in their exclusion from the analyses. Missing data for the included components was replaced by the mean value of observed similar foods. Thus, the nutrient content of food may be one of the distinguishing factors for the traditional Chinese medicine categorisation of the cold-hot nature of foods. These papers exemplify exploring nutrient differences between generally accepted food categories and informs the metabolic effect of consumption from these categories.

Ispirova et al. [21] aimed to address the issue of missing data in FCDBs by using imputation techniques based on statistical prediction. The four imputation methods applied were non-negative matrix factorization (NMF), multiple imputations by chained equations (MICE), nonparametric missing value imputation using random forest (MissForest), and k-nearest neighbours (KNN). These methods were compared with the traditional approaches of fill-in with the mean and fill-in with the median. Two types of datasets were also considered. The first type explored imputing values for the same nutrient in one food item using different national FCDBs. The second type explored imputing nutrient values from the same FCDB using similar food items. Using five regression metrics to assess the performance of the imputation techniques, the study found that the commonly used approaches always resulted in the largest errors. For all the statistical prediction techniques, the error increased with the percentage of missing data. Overall, the MissForest imputation method performed best by yielding the smallest errors. It is necessary to note that imputation using random forests is distribution-free or non-parametric. This makes it more flexible than the distribution-based methods and is more suitable for use when the data contains non-linearities or interactions.

### 3.3. Clustering

After standard statistical methods, clustering was the next most utilized statistical method. Cluster analysis is a class of multivariate methods that aim to classify observations into homogenous groups that are different from other groups. Seven papers applied clustering methods to FCDBs. Windham et al. [36] applied the fuzzy c-means clustering algorithm within the dairy, grain, and fat commodity groups to determine foods with a similar nutritional content. The authors opted to classify foods both by nutrients that are scarce in terms of food supply and by nutrients that pose health risks when consumed in excess, thus expanding current groupings.

Akbay et al. [15] aimed to divide lamb meat into compositionally similar groups to offer healthier dietary replacements. The composition data of several lamb preparations was extracted from the FCDB and analysed using agglomerative hierarchical cluster analysis with average linkage. The Euclidean distance was used, and the data was standardised. The results found two distinct clusters (with families and subfamilies) differing in fatty acids, cholesterol, and energy. This distinction is beneficial to the population in general in making informed dietary choices.

Pennington and Fisher [29] aimed to empirically group fruits and vegetables based on food components of public health significance and thereafter, relate them to four classification variables: botanic family, colour, part of the plant, and total antioxidant capacity. The proposed classifications may aid researchers and nutritionists in developing FFQs and providing dietary guidance. The data consisted of food composition information for 37 fruits and 67 vegetables. Missing values were obtained from the literature or imputed. An agglomerative hierarchical clustering method using Ward’s linkage was applied to 23 food components, which were standardised using the range method. Graphical (dendrograms) and statistical (pseudo t^2^ statistic) methods were used to determine the optimal number of clusters and multivariate analysis of variance (MANOVA) was used to compare differences between cluster means for each of the 23 food components. The study mentioned that while cluster membership differed by cluster methodology, certain fruits and vegetables remained grouped, suggesting value in employing mathematical clustering as a guide toward useful groupings.

A more recent study was documented by do Prado et al. [32] in Brazil. Manufactured food products are often reformulated to reduce the content of negative nutrients, such as sodium, or to increase the content of positive nutrients, such as dietary fibre. This study aimed to compare the change in the nutrient composition of specific Brazilian food groups (259 food items) from 2003 and 2013 using percentage change, hierarchical cluster analysis (HCA), and principal component analysis (PCA). The Brazilian FCDB was used for 2003. Analytical reports and updated data from food manufacturers were used for 2013. Percentage change in component data was classified as either negligible change (0–9.99%), moderate reformulation (10–24.99%), or substantial reformulation (≥25%). HCA was used to separate food items within each food group (cereal and cereal products, meat and meat products, milk and milk products, and manufactured foods) based on their compositional similarities. The results showed that some food items did not cluster together despite having similar matrices. This suggests that using pre-established food groups alone may be inaccurate in assessing changes in nutritional composition and should be combined with multivariate statistical analyses. PCA was also used to partition food items within food groups. After comparison with the HCA results, HCA was found to be the most suitable tool to group the food products concerning their composition. Mean nutrient content for 2003 and 2013 was calculated for each HCA-derived cluster and compared using a paired Student’s *t*-test or Wilcoxon test. The percentage change for each cluster was also calculated. do Prado et al. [32] concluded that the joint use of percentage change and other statistical techniques allowed efficient identification of changes in the nutritional composition of food items.

Another study that used both PCA and HCA was conducted by Li et al. [24]. Li et al. [24] analysed 268 raw plant foods from 5 food categories. Applying a PCA showed that cereal grains, nuts and seeds, and legumes could be well separated when considering nutritional content, but fruits and vegetables exhibited significant overlap. A follow-up soft independent modelling of class analogies (SIMCA) analysis suggested that nuts and seeds are similar to all other plants. Finally, using agglomerative hierarchical clustering with Ward’s distance, the resulting clusters contained foods from different food categories. Better separation was achieved using clusters based on compositional similarity rather than the food categories.

Atsa’am et al. [16] applied k-means clustering with Euclidean distance to food items within the ‘cereals’ category of the West African Food Composition Table. Thirteen nutrients were analysed, and the data utilised a min-max normalisation. The within-group and between-group sum of squares was used to validate the clusters found. The extracted clusters separated the food items by the type of grain and preparation method. For example, all millet items occupied its own cluster and boiled grains were distinct from their raw counterparts.

Nikitina et al. [28] examined 330 cottage cheese products and confectionaries and aimed to cluster them by their carbohydrate content using k-means clustering. The algorithm identified clusters that grouped foods as having either a low, medium, or high carbohydrate content. These clusters are useful for diet planning for diabetics.

### 3.4. Dimension Reduction Techniques

PCA [41] and factor analysis [42] are data reduction techniques that combine correlated variables into a smaller number of components. Two studies [17,33] investigated the nutrient co-occurrence patterns in foods. Similä et al. [33] used the food composition database of Finland, which consisted of 530 food items, used as ingredients (fresh, uncooked, and edible), and 106 nutrients and non-nutrients (for instance, phytosterols and heavy metals). The analysis method was factor analysis with varimax rotation and was applied to two approaches of the data. The first approach used nutrient values per 100 g of foods whilst the second approach used nutrient values in a portion of 1 MJ of each food (nutrient densities). The nutrient content patterns identified for both approaches were easily interpretable and consistent with prior knowledge of nutrient composition.

Balakrishna et al. [17] also investigated nutrient co-occurrence patterns. PCA with varimax rotation was applied to 971 food items and 28 nutrients from the South African FCDB. PCA was applied to the nutrients, to establish nutrient patterns, and to the food items themselves for validation. The nutrient patterns obtained mirrored the South African food-based dietary guidelines (FBDGs). One of the patterns identified foods that had a high sodium content, and this corresponded with foods identified under the country’s national salt regulations. FBDGs recommend the consumption of foods instead of nutrients and changes in food consumption changes the intake of several nutrients, not just one. Thus, information on nutrient co-occurrence patterns within foods is needed.

### 3.5. Other Methods

One paper [34] described applying linear programming (LP) and quadratic programming (QP) methods to FCDBs. LP and QP are processes that find optimal solutions to an objective function subject to one or more constraints. FCDBs often contain information for many nutrients but have values for only a small number since chemical analyses are costly. With the many products being introduced each year, obtaining these missing values via chemical analyses is impractical. Calculation of these missing values can be achieved if a food is a composite of other foods in the database and one knows the proportions of the ingredients and the method of preparation. Other methods using food labels are available but can be time-consuming. Thus, Westrich et al. [34] aimed to develop a mathematical optimisation software that estimates unknown nutrient estimates in products. The optimisation software was able to estimate nutrient values four times faster than conventional (trial-and-error) methods with the same degree of accuracy, although the QP method was slightly slower than LP. The LP version of the software was adopted to help maintain the database.

Ispirova et al. [20] also investigated the missing data problem. Traditionally, missing nutrient values are borrowed from other countries. However, we must be cognizant of the different geographical elements between countries and differing qualities of FCDBs. In practice, borrowing is achieved using the FCDB of a neighbouring country or from that which has a large span of data. The authors developed a method to objectively, rather than subjectively, borrow nutrient values using null hypothesis testing. After ensuring the similarity in the method type (analytical values were used) and food items between the FCDBs of 10 countries, the value for a specific nutrient and food item was compared across the FCDBs using the appropriate statistical tests and post hoc procedures, e.g., Friedman test with Nemenyi and Holm post hoc procedures. This methodology (Missing Nutrient Value Imputation UsinG Null Hypothesis Testing—MIGHT) gave more accurate results for imputation when compared to currently used techniques. MIGHT supports the premise that proper statistical analysis can improve the missing data problem of FCDBs while minimising the error of current nutrient imputation practices.

Phanich et al. [31] applied the Self-Organising Map (SOM) and k-means clustering to Thai food composition data to develop a food recommendation system for diabetic patients. This system enabled diabetic patients to find suitable substitutions, similar in the nutrient composition and characteristics, for food items. The SOM, or Kohonen network, is a type of artificial neural network used for the visualisation and analysis of high-dimensional data. The data is described by a finite set of models, which are associated with neurons or nodes in the network. The closer the nodes in the network, the more similar the models. The dataset consisted of 290 Thai food dishes and 8 nutritionist-recommended nutrients that influence diabetes. Food items were grouped by their characteristics (such as noodles, rice, and fried food) and were also grouped by a nutritionist into normal food, limited food, and avoidable food based on dietary recommendations for diabetics. The two-stage analysis first constructed and trained the SOM before clustering the SOM using the k-means approach. The resulting clusters contained foods that provided similar amounts of the eight nutrients. The system scored well amongst the nutritionists who were invited to evaluate it.

Kim et al. [23] employed network-based approaches in their analysis of FCDBs. Network-based approaches and average linkage hierarchical clustering methods were applied to consolidate foods that had almost identical nutrient contents, and then to connect foods with similar nutrient contents. The aim was to determine the nutritional balance of food items to aid the design of healthy diets. Many individual nutrients were identified that instrumentally contributed to the nutritional balance. However, pairs of nutrients could also impact the nutritional balance of food while the individual nutrients alone may not. This additional complexity was quantified using Pearson correlations between nutrients (across foods). The network-based approach provided an overview of the relationships between foods and food groups and the relationships between nutrients.

## 4. Discussion

**Figure 2:**
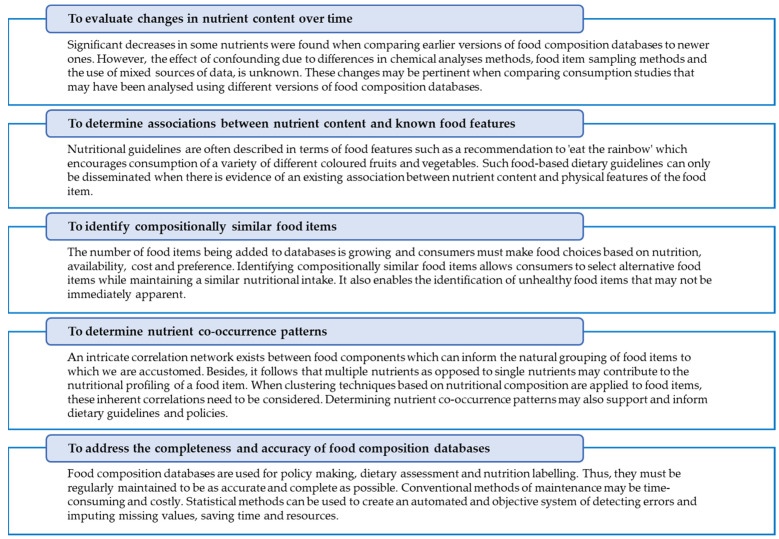
Themes from the literature on the application of statistical methods to food composition data.

This review set out to provide coverage and depth of published studies that applied statistical methods to food composition databases. The results indicate that the number of studies in the literature utilising statistical methods for the analysis of food composition databases has increased over the years. Providing a summary of the literature and highlighting areas for further development, that could guide future studies in the statistical analysis of food composition databases, could be helpful. The review has identified five objectives of statistical analyses that have been applied to FCD and these are summarised in Figure 2.

Associating nutrients with known food characteristics was a common aim among the included studies. These characteristics could be physical such as colour or part of a plant [29,30], conceptual such as the cold- and hot-nature assignment in traditional Chinese medicine [25,37], or they could be existing food labels such as ‘fat-free’ and ‘low-fat’ [27]. Establishing the relationship between the composition and characteristic with statistical analysis provides strong evidence to support dietary recommendations and guidelines. Dietary choices become more accessible to consumers and can also confirm prior knowledge of nutrient composition. The nutrient content of a food characteristic-based diet such as the ‘rainbow’ diet [43] can be ascertained and specific nutrient guidelines can be provided to the wider audience in the form of easily recognisable food features. Statistical methods used to determine associations between food features and nutrient composition were generally standard statistical methods and regression methods. Analysing differences in the mean nutritional content between foods grouped by features can describe the associations. The assumption that different groupings of food items have unique concentrations of nutrients and minerals is a fundamental concept for using outlier detection to detect errors in food composition data, as shown in [18]. Alternatively, regression models are easy to understand and use for prediction and can include all explanatory variables that are of interest. However, users need to be aware of poor data quality and collinearity among the predictors as these issues can affect the validity and predictive power of the model. As shown in [9,17,24], particular nutrients can be highly correlated and could contribute to collinearity. The reliability of the model can also decrease with an increasing number of predictors. More advanced regression methods such as ridge and LASSO regression [44] may be needed in cases where one has multicollinearity or a large number of explanatory variables. Random forests [45] can also be used to determine variable importance. Regression methods are often based on certain assumptions, which must be tested using diagnostic techniques.

Clustering also featured prominently among the studies. Clustering techniques were used to separate food items into distinct groups based on their nutritional profile. This enabled the identification of foods that were similar in nutrient composition and thus offered alternatives that were either healthier or more readily available. Clustering also suggested that food groups based on compositional similarity might be different from FCDB categories. Four common clustering techniques are centroid-based, density-based, distribution-based, and hierarchical clustering. A comprehensive review of available clustering methods can be found in [46]. Most studies implemented k-means clustering (centroid-based) and hierarchical clustering, suggesting that opportunities for future research in applying density- and distribution-based clustering exist. In general, clustering is intuitive and identifying distinct subgroups can be beneficial in making sense of large datasets. However, certain elements of clustering algorithms can introduce subjectivity such as the choice of clustering algorithm, similarity measure, number of clusters, and initial values. Clustering can also be classified as ‘hard’ or ‘soft’. With hard-clustering methods such as k-means and hierarchical clustering, food items are assigned to only one cluster. Measures of similarity and distance such as Euclidean distance or Mahalanobis distance are often used, and the choice of the measure could affect the number of clusters generated from the data. In contrast, employing soft-clustering methods, such as fuzzy and distribution-based clustering, assigns food items to groups using probability means; thus, a unit could be assigned into more than one group. Only one study [36] employed a soft-clustering method. Employing soft-clustering methods for the analysis of FCD is appropriate when delimits between distinct food groups are unclear and further applications of soft-clustering methods are recommended to determine any advantages. The categorisation of food items into conceptually and compositionally similar groups allows the offer of healthier dietary choices by creating data-driven exchange lists. Identifying compositionally similar foods allows individuals to exchange foods to meet health requirements, financial constraints, or personal preferences. Unhealthier manufactured foods that are not immediately apparent can also be identified through clustering techniques. Thus, improving multivariate techniques for food composition data and its applications has a far-reaching impact.

An important area of investigation to improve multivariate techniques is the correlation and co-occurrence of food components. Correlation methods and network-based approaches capture the intimate correlations that exist between nutrients, which contributes to the partitioning of food items into distinct categories. This needs to be accounted for in the clustering methods employed and other statistical techniques such as regression methods. PCA and factor analysis were used to investigate nutrient co-occurrence patterns amongst food items. One study was able to relate the nutrient patterns found to existing national guidelines and policies [17]. The advantages of using factor analysis and PCA to analyse FCD are that several nutrients are considered at once and the components found can be used as covariates in models when the use of the original larger dataset is not feasible. Some disadvantages include the subjective selection of the number of components and the subjective determination of which variables are important to a component. The interpretability of the components can also be difficult. Lastly, since it is common practice to select only some of the components, the components that are excluded may result in information loss [5]. As more food items are added to a database, additional groupings may be needed to describe the variability in the food components. These techniques may be able to reduce this high-dimensional data to a few patterns that capture the variation.

Detecting changes in food composition was also found to be a theme among the included studies, thus highlighting the impact on food consumption studies. Nutrient database revisions are necessary to maintain the accuracy of the values obtained from improved chemical analytical techniques and sampling methods. Updates can also reflect product reformulation and fortification. The significant differences found between different versions of a database impacts on comparisons drawn between consumption studies over time. The guidelines formulated based on the previous nutrient content of food items may also need to be adjusted to reflect these changes. The included studies mostly used standard statistical methods to detect changes in nutrient composition; however, its combination with clustering methods may be more useful [32].

FCDBs face problems of incorrect and missing nutrient values. The imputation of missing food composition data has recently surfaced to make FCDBs as accurate and complete as possible. Missing data biases results as food items or components with a large proportion of missingness are often excluded from the analysis. Imputation can be carried out to prevent loss of information, but the method of imputation needs to be carefully considered. Commonly used approaches such as fill-in with mean have been shown to result in large errors when compared to more sophisticated machine learning techniques [21]. Optimisation and hypothesis testing techniques were also used to increase the quality of FCDBs [20,34] and the accuracy of estimating missing values was increased while being faster than conventional methods (e.g., trial-and-error or borrowing), which can be subjective and prone to error. For imputation, the advantages of using the optimisation methods are that a structured model of the problem can be created, the objectivity of the results is increased, complex problems that have many decision variables and constraints become easier to solve, and solutions can be re-evaluated if decision variables change. However, disadvantages include the incorrect modelling of complex problems, and the applicability of the mathematical solution to a real-life problem [47]. In addition, Ispirova et al. [20] demonstrated how statistical methods combined with the traditional practice of borrowing data from other FCDBs can result in a more objective and accurate imputation. The missing data imputation method needs to incorporate the mechanism of missingness (missing at random, missing completely at random, or missing not at random) and the type of imputation needed (using other databases or using the same FCDB). Advancing statistical methods to account for these issues is needed.

Both the variety of food consumed by a population and the nutritional composition of food differs by geographic location. The former is affected by culture and availability whilst the latter is affected by environmental, genetic, and processing influences. Hence, an important part of understanding the data and results is knowing the source of the data. Only two of the included studies used data from Africa while the majority used data from the United States of America, Europe, and Asia. Not only will the foods consumed in high-income countries differ from the foods consumed in low- and middle-income countries but nutrient composition and content patterns established in high-income countries will possibly vary from that of low- and middle-income countries as well [48]. There is an opportunity to explore nutrient content patterns amongst locally available food items and compare these with patterns from other countries. FCD is available for many countries; yet, the analysis of this data remains largely unexplored, possibly due to a lack of information regarding research potential and appropriate analysis methods. By gathering the research trends and analysis methods used, opportunities for future research will be made available to countries with untapped data.

This literature review has some limitations. For practical reasons, only publications that had an English translation available were included in the review, thus subjecting the review to a possible language bias. However, only two studies were excluded from the full-text review for this reason. Moreover, the selection and data extraction of studies were carried out by one author. However, inclusion and exclusion criteria were developed to clarify the systematic process of selection. Although the quality of the studies was not assessed, the focus of the review was on the type of statistical method used and its objective rather than the results of individual studies. Thus, any bias in the results of the included studies, arising from inappropriate study methodology, is minimal.

It should be noted that many studies relating to nutritional profiling (NP) [49] were found. Studies with the objective of nutrient profiling were excluded. While many of these studies used statistical methods, they also required the input of extensive secondary data such as recommended daily allowances and knowledge of dietary guidelines. Hence, we believe that NP models fall outside the scope of this review and should be considered independently of other statistical methods applied to FCDBs.

## 5. Conclusions

To our knowledge, this is the first review of the literature looking at statistical methods applied to FCDBs. Minimal literature from Africa was found, lending the opportunity for the continent to contribute to this vital area by analysing their own unique food patterns. Many prospects for the combined use of FCDBs and statistical methods exist. To begin, an exploratory data analysis using standard statistical methods to understand the behaviour of the data and to describe the existing relationships between the nutrients and food groups is recommended. FCDBs are large datasets that are continuously expanding due to the addition of new food items and newly available nutrient values. Thus, they will also benefit from the application of dimension reduction techniques to establish nutrient patterns and to facilitate further research that may require a reduced dataset. Clustering techniques, especially soft-clustering techniques, will identify nutritionally similar food items and could provide data-driven results of latent discriminating factors. Associating nutritional value with easily identifiable food characteristics is important for informing nutrition and dietary practice tools, such as food exchange lists and FBDGs, which can be achieved using regression methods. Since these tools guide nutrition research, practice, and nutrition education, it is imperative that they are as complete and accurate as possible. Several imputation options using advanced statistical techniques, such as regression, hypothesis testing, and optimisation, can be used by FCDB compilers to maintain and improve their data. Finally, FCD has unique characteristics such as correlated components, natural groupings, and a compositional nature. The choice of statistical method used needs to account for this data structure. As further research, we are using modern clustering methods to identify groups of foods that are similar in macronutrient, mineral, and vitamin content. This will enable nutritionists to consider multiple food components simultaneously to objectively and comprehensively classify food. Statistical methods can handle multiple variables, extract latent features, objectively classify food items, and efficiently impute missing values. Innovative statistical methods tailored towards food composition data may provide improved evidence for consumption study results, dietary advice, guidelines, and policies.

## Figures and Tables

**Table 1:** Description of included studies.

Study	Year	Country of Data	Objectives	Methods	Main results
Akbay et al. [15]	2000	United States of America	To divide lamb meat into groups distinct in dietary fat and associated nutrients, to offer healthier dietary replacements.	Agglomerative hierarchical cluster analysis with average linkage	Two main clusters were found in the lamb meat data. One of the clusters divided into two families and four subfamilies based on fatty acids, cholesterol, and energy composition.
Atsa’am et al. [16]	2021	West Africa	To determine subgroupings within the ‘cereals’ category of foods.	K-means clustering with Euclidean distance	Six subgroups within the ‘cereals’ category were found which separated the grains by type and preparation method.
Balakrishna et al. [17]	2021	South Africa	To determine nutrient co-occurrence patterns and compositionally similar food groupings.	Spearman’s rank correlation, principal component analysis	Significant correlations were found among the nutrients. Eight nutrient patterns were obtained, which mirrored the South African food-based dietary guidelines.
Chu et al. [18]	2009	Taiwan	To detect unlikely nutrient values (errors) in a food composition database.	Ranking coefficients of variation for each nutrient by food subgroup and detecting outliers	Compared to manual assessment, error detection increased 38-fold with the computerised process.
Davis et al. [19]	2004	United States of America	To determine possible changes in nutrient composition for garden crops between 1950 and 1990.	Wilcoxon signed-rank test	Of the 13 nutrients analysed for 43 food items, 6 exhibited statistically significant declines from 1950 to 1990. Declines ranged from 6% to 38%.
Ispirova et al. [20]	2019	Italy, United Kingdom, Switzerland, Sweden, Slovenia, Belgium, Denmark, Netherlands, United States of America, Canada	To decrease the error of data borrowing when imputing missing nutrient values from other food composition databases.	Non-negative matrix factorization, null hypothesis testing	When borrowing from other food composition databases, the proposed methodology produced smaller absolute errors more often than regular borrowing methods.
Ispirova et al. [21]	2020	Italy, United Kingdom, Switzerland, Sweden, Slovenia, Belgium, Denmark, The Netherlands, United States of America, Canada	To evaluate imputation methods for missing data in food composition databases.	Non-negative matrix factorization, multiple imputations by chained equations, nonparametric missing value imputation using random forest, k-nearest neighbour	Imputation methods using statistical prediction performed better than traditional approaches of fill-in with the mean and fill-in with the median. Overall, the missing value imputation using the random forest technique performed the best by yielding the smallest error.
Khan [22]	1996	Australia	To identify thresholds that would rank food items according to a low, medium, or high nutrient content.	Measures of central tendency	No optimum ranking scheme was recommended, but guidelines were provided. Three different criteria were deemed suitable in line with the proposed guidelines.
Kim et al. [23]	2015	United States of America	To investigate the relationships between food items and between nutrients to inform nutrition.	Network-based approaches, hierarchical clustering with average linkage, pairwise correlations	Clustering revealed a hierarchical organisation of food that was consistent with common nutritional knowledge but also found unexpected relationships between food items. Similarly, significant positive pairwise correlations were found to exist between nutrients.
Li et al. [24]	2021	United States of America	To categorise raw plant foods according to nutritional similarity.	Spearman’s rank correlation, principal component analysis, soft independent modelling of class analogies (SIMCA), agglomerative hierarchical clustering	Four clusters were identified that consisted of foods from different food groups. Better separation was achieved using clusters rather than traditional food groups.
Liu et al. [25]	2012	China	All foods in traditional Chinese medicine are categorised into ‘the four natures’: cold, cool, warm, and hot. The purpose of this paper is to examine the association between the nutrient content of these foods and their cold-hot nature category.	Logistic regression	Fat, carbohydrate, and selenium were significantly associated with the hot nature of foods while iron and copper were significantly associated with the cold nature of foods. The results suggest that the nutrient contents of foods may be one of the distinguishing factors for the categorisation of the cold-hot nature of foods.
Mayer [26]	1997	United Kingdom	To determine if the nutritional composition of fruits and vegetables had changed between the 1930s and 1980s.	Student’s *t*-test	There was a reduction in the nutrient content for several food items over the 50 years.
Nguyen et al. [27]	2016	United States of America	To determine whether ‘healthier’ versions of common foods have more sugar than ‘regular’ counterparts.	Friedman test, post-hoc Wilcoxon signed-rank test	The sugar content of foods classified as ‘low-fat’ and ‘non-fat’ was higher than that of regular versions.
Nikitina et al. [28]	2021	Unknown	To cluster cottage cheese products and confectionary by carbohydrate content.	K-means clustering	Five clusters were found which identified foods with low, medium, and high carbohydrate contents.
Pennington and Fisher [29]	2009	United States of America	To empirically group fruits and vegetables based on food components of public health significance and thereafter, relate them to four classification variables: botanic family, colour, part of the plant, and total antioxidant capacity.	Agglomerative hierarchical clustering with Ward’s linkage, multivariate analysis of variance (MANOVA)	Eight clusters were identified that could be used to classify the 104 fruits and vegetables. Clusters were best defined by a combination of classification variables, such as colour and part of the plant, and were predictive of the nutritional profile.
Pennington and Fisher [30]	2010	United States of America	To determine fruit and vegetable subgroups with significantly higher concentrations of 24 food components.	Kruskal–Wallis, one-way analysis of variance (ANOVA)	Concentrations of the 24 food components differed between the subgroups and can be used to aid nutritional guidelines.
Phanich et al. [31]	2010	Thailand	To develop a food recommendation system for diabetic patients enabling diabetic patients to find suitable substitutions, similar in nutrient composition and characteristics, for food items.	Self-Organising Map (SOM), k-means clustering	The resulting clusters contained foods that provided similar amounts of eight selected nutrients. Patients were able to use the developed software to select healthier alternatives to current food choices.
do Prado et al. [32]	2016	Brazil	To compare the change in the nutrient composition of specific Brazilian food groups between 2003 and 2013.	Percentage change, hierarchical cluster analysis, principal component analysis	The results showed that using pre-established food groups alone may be inaccurate in assessing changes in nutritional composition. Hierarchical cluster analysis combined with percentage change allowed efficient identification of the changes in the nutritional composition of food items.
Similä et al. [33]	2006	Finland	To obtain information about nutrient co-occurrence patterns among food items.	Factor analysis	Four nutrient content patterns, which was consistent with prior knowledge, were identified. The patterns were characterised by (1) fish, meat, dairy products, legumes, seeds, and nuts; (2) vegetable fats; (3) staple foods; and (4) offal foods (liver, kidney).
Westrich et al. [34]	1998	United States of America	To estimate unknown nutrient values in commercial food products.	Linear programming optimisation, quadratic programming optimisation	The proposed optimisation software was able to estimate missing nutrient values four times faster than conventional methods with the same degree of accuracy. The linear programming method was found to be faster than the quadratic programming method.
White and Broadley [35]	2005	United Kingdom, United States of America	To determine nutrient composition changes in fruits and vegetables between the 1930s and 1980s.	Student’s *t*-test	Average concentrations of certain minerals in fruits and vegetables had significantly decreased between the 1930s and 1980s.
Windham et al. [36]	1985	United States of America	To group foods within the dairy, grain, and fat commodity groups depending on nutrients of current concern.	Fuzzy c-means clustering	Five clusters within each commodity group were identified. Nutrient content was similar within subgroups and overcame the problem of objective and accurate grouping when dealing simultaneously with many nutrients. The use of fuzzy clustering avoided the use of arbitrary cut-offs when determining foods high and low in specific nutrients.
Xie et al. [37]	2020	United States of America, China	To determine the nutrients affecting the cold-hot nature property of food items as per traditional Chinese medicine.	Multivariate ordinal regression, ANOVA	Six components (folate, vitamin B6, calcium, vitamin A, and caffeine) were found to be predictive of the cold, plain, and hot nature of foods.
Yarbrough Al-Bander et al. [38]	1988	United Kingdom	To explore the distribution of chloride content in uncooked foods and determine its correlation with sodium content.	Wilcoxon signed-rank test, linear regression	Chloride and sodium content exhibited large variation among the 216 uncooked food items. Chloride and sodium content were strongly correlated.

**Table 2:** Characteristics of included studies (*n* = 24).

Characteristic	*n*	%
Primary statistical method		
Standard statistical methods ^a^	8	33.3
Regression methods	3	12.5
Clustering	7	29.1
Dimension reduction techniques	2	8.3
Other ^b^	4	16.7
Aims ^c^		
To evaluate changes in nutrient content over time	4	16.7
To determine associations between nutrient content and known food features	6	25.0
To identify compositionally similar food items	9	37.5
To determine nutrient co-occurrence patterns	5	20.8
To address the completeness and accuracy of food composition databases	4	16.7
Country of data used ^c^		
United States of America	13	54.2
United Kingdom	5	20.8
Other ^d^	11	45.8

^a^ Descriptive statistics, Student’s *t*-test, Wilcoxon signed-rank, Kruskal–Wallis test, and Friedman test. ^b^ Network-based approaches, mathematical optimisation, and hypothesis testing. ^c^ Some studies addressed more than one aim or used data from more than one country. One study did not specify the country of data used. ^d^ Australia, Belgium, Brazil, Canada, China, Denmark, Finland, Italy, The Netherlands, Slovenia, South Africa, Sweden, Switzerland, Taiwan, Thailand, and West Africa.

## Data Availability

No new data were created or analysed in this study. Data sharing is not applicable to this article.
